# Recent Advances in Cellular Glycomic Analyses

**DOI:** 10.3390/biom3010198

**Published:** 2013-02-21

**Authors:** Jun-ichi Furukawa, Naoki Fujitani, Yasuro Shinohara

**Affiliations:** Laboratory of Medical and Functional Glycomics, Graduate School of Advanced Life Science and Frontier Research Center for Post-Genome Science and Technology, Hokkaido University, Sapporo 001-0021, Japan: jfuru@sci.hokudai.ac.jp (J.F.); fujitaninaoki@sci.hokudai.ac.jp (N.F.)

**Keywords:** cellular glycomics, *N*-glycans, *O*-glycans, glycosphingolipids, mass spectrometry, glycosaminoglycans, sample preparation

## Abstract

A large variety of glycans is intricately located on the cell surface, and the overall profile (the glycome, given the entire repertoire of glycoconjugate-associated sugars in cells and tissues) is believed to be crucial for the diverse roles of glycans, which are mediated by specific interactions that control cell-cell adhesion, immune response, microbial pathogenesis and other cellular events. The glycomic profile also reflects cellular alterations, such as development, differentiation and cancerous change. A glycoconjugate-based approach would therefore be expected to streamline discovery of novel cellular biomarkers. Development of such an approach has proven challenging, due to the technical difficulties associated with the analysis of various types of cellular glycomes; however, recent progress in the development of analytical methodologies and strategies has begun to clarify the cellular glycomics of various classes of glycoconjugates. This review focuses on recent advances in the technical aspects of cellular glycomic analyses of major classes of glycoconjugates, including *N*- and *O*-linked glycans, derived from glycoproteins, proteoglycans and glycosphingolipids. Articles that unveil the glycomics of various biologically important cells, including embryonic and somatic stem cells, induced pluripotent stem (iPS) cells and cancer cells, are discussed.

## 1. Introduction

Human embryonic stem (ES) cells derived from the inner cell mass of the blastocyst and induced pluripotent stem (iPS) cells reprogrammed from somatic cells display indefinite growth, while maintaining pluripotency [1,2]. Extensive research has been conducted with these cells to help develop disease models, methods for drug screening and regenerative therapies. As progress using stem-cell-driven therapy has evolved dramatically, effective quality control methods and standardization of human iPS cells have become important requirements for clinical application of these therapies. The significance of identifying highly validated cellular biomarkers that enable prediction of health conditions and screening of patients is also evident. The Critical Path Initiative of the US Food and Drug Administration (FDA) highlights the importance of biomarkers that can predict or monitor responses to therapy for *in vitro* diagnostics, imaging and preclinical toxicogenomics [3]. Cells are currently defined by a combination of physical, phenotypic and functional properties; the identification of novel cell surface markers is highly advantageous to the rapid detection, characterization and isolation of particular cell populations. In fact, many studies aimed at discovering novel cellular biomarkers based on gene expression, proteomics and metabolite analyses are currently in progress [4].

Glycan expression analysis is an attractive option for the development of novel cellular biomarkers, because many of the frequently employed biomarkers, such as stage-specific embryonic antigens (SSEA-3/4/5) and tumor-rejection antigens (Tra-1-60 and Tra-1-81), are glycoconjugates [5,6,7,8,9]. Most FDA-approved tumor markers are either glycans or glycoproteins (e.g., AFP, CA10-9, CA125, CEA, PSA and HER2/NEU) [10]. These glycomarkers were identified following the rather fortuitous development of specific anti-glycoconjugate antibodies. The glycome is indirectly related to the genome through the specificity of glycosyltransferases that perform non-template mediated biosynthesis of glycans. By regulating the expression of glycan degradation enzymes and supply of nucleotide-activated sugars, a cell can produce glycan structures that are distinct from those of neighboring cell types. The glycans produced by each cell are a highly heterogeneous nested set of related structures that result from alternative branching patterns, incomplete glycosylation and post-glycosylational modifications, such as sulfation and acetylation [11]. These variations produce an enormous number of biosynthetically permissible glycan structures. The size of the cellular glycome is a matter of debate, but the number of glycan structures is estimated to be in excess of 100,000 to 500,000 [12]. Due to these complexities, direct analysis of glycans is highly valuable for accurate clarification of the spectra of cellular glycomics. Additional research into the cellular glycome may help to identify a potential goldmine of biomarkers.

Contrary to linear DNA and protein sequences, glycans have heterogeneous structures that differ in composition, branching, linkage and anomericity. These differences have caused glycomic research to lag far behind DNA- and protein-based research. However, rapid growth and interest in glycomic research, along with improved mass spectrometric (MS) analytical methodologies, have begun to aid development of powerful analytical procedures that meet the demand for cellular glycomics. 

Cell surfaces are coated with a variety of intricately arranged glycoconjugates. Major components of the cellular glycome include *N*- and *O*-linked glycans derived from glycoproteins, glycosaminoglycans and glycosphingolipids. It is widely appreciated that the relative positioning of glycans, which can be regulated by their display on glycoprotein scaffolds with three-dimensional geometries or the spatial arrangement of glycoconjugates, can profoundly influence the avidity and specificity of glycan-oligomerized receptor interactions [13]. In addition, some carbohydrate epitopes (e.g., Lewis^x^, also known as SSEA-1) are known to be constituents of different glycoconjugates [14]. Therefore, it is important to clarify the distribution of particular epitopes in a cross-glycomics manner. Transgenic and knockout mice in which various glycogenes were removed or altered had no apparent phenotype [15], suggesting that compensation by alternate glycogene(s) may occur. These results indicate the importance of understanding the role of intra- and inter-glycomic correlations (correlations within and between the individual glycomes of various glycoconjugates) in maintaining cellular homeostasis and response to changes. Therefore, it is important to delineate a comprehensive cellular glycome by analysis of all major glycoconjugates, rather than individual glycomes. In this review, we will discuss structurally intensive methods that focus on the glycan moiety and enable analysis of the elemental glycomics of *N*- and *O*-linked glycans derived from glycoproteins, proteoglycans and glycosphingolipids, which are the major components of cell surface glycoconjugates.

## 2. Recent Advances in Elementary Glycomic Analysis

### 2.1. *N*-Glycans

Protein *N*-glycosylations play important roles in folding, oligomerization, sorting and transport of proteins [16,17]. De-*N*-glycosylation by peptide *N*-glycosidase F (PNGase F), and subsequent purification and detection protocols are well established; therefore, the cellular *N*-glycome is the most routinely analyzed glycome among various classes of glycoconjugates. In the Human Proteome Organization’s human disease glycomics/proteomics initiative project, the relative abundance of various *N*-glycans in a number of glycoprotein samples was analyzed in 20 laboratories, and the results of chromatographic and MS analyses were evaluated [18]. Although it is generally accepted that MS is not capable of accurately quantifying oligosaccharides unless stable isotope-labeled analogs are incorporated as internal standards, the results of this multi-institutional study indicated that matrix-assisted laser desorption/ionization (MALDI) time-of-flight MS and liquid chromatography (LC)/electrospray ionization (ESI) MS yielded quite compatible results with chromatography in the quantitation of oligosaccharides. A comparable study of the signal intensities generated from a mixture of equimolar amounts of various underivatized *N*-glycans in the MALDI mass spectrum did not show any significance for compounds with molecular masses greater than approximately 1 kDa [19]. It was also reported that methyl esterification of sialic acid renders sialylated oligosaccharides that are chemically equivalent to neutral oligosaccharides and allows simultaneous analysis of neutral and sialylated oligosaccharides by MALDI-TOF MS [20,21]. The inherently rapid and accurate nature of detection by MALDI-TOF MS gives this approach unique and significant advantages for high-throughput glycomic analysis [22]. 

Considering that quantitative cellular *N*-glycomics relies on highly efficient and reproducible liberation of oligosaccharides from cellular glycoproteins, it is important to maximize the efficiency of *N*-glycan liberation. Glycoproteins differ widely in their susceptibility to enzymatic digestion, since glycosylated sites are often obstructed by secondary and tertiary protein structures. Therefore, differences in the conditions of de-*N*-glycosylation by PNGase F may cause alterations in liberation efficiency. For example, preparation of tryptic glycopeptides and reductive alkylation facilitate the release of *N*-glycans via PNGase F digestion [23]. 

Upon liberation, *N*-glycans are analyzed in their intact form, reduced form, permethylated form or other derivatives used for reducing end modifications [24,25]. Permethylation of glycan hydroxyl groups has been used to facilitate sample clean-up; increase glycan hydrophobicity and volatility, which translates into higher sensitivity during MS analysis; allow simultaneous analysis of neutral and sialylated oligosaccharides; and facilitate MS/MS analysis by leading to predictable fragmentation. Miniaturized approaches using micro-spin columns packed with NaOH that permit effective derivatization with methyl iodide in less than one minute have also been described [26]. High-throughput quantitative analysis of *N*-glycans by MALDI-TOF MS has been demonstrated by using prior solid-phase permethylation in a 96-well plate configuration [27]. The lack of hydroxyl groups prevents the cleavage of other glycosidic bonds, rendering the permethylated oligosaccharides resistant to in-source fragmentation [28]. To confirm glycan composition, as well as obtain detailed structural information, selected ions are often subjected to tandem mass spectrometry (e.g., TOF/TOF). To define monosaccharides and their anomeric configurations and to confirm tentative sequences, glycans are often treated with exoglycosidases. These enzymes are specific to the stereochemical and anomeric configurations of the monosaccharides, including α2-6-, α2-3- and α2-8-bound sialic acid; α1-2-, α1-3/4- and α1-6-bound fucose; and β1-3- and β1-4-bound galactose. 

With the advent of MALDI-TOF MS technology and the basic research described above, it has become possible to define the structures of the multitude of individual glycans in various cell lines. A number of glycomic studies clarifying cellular *N*-glycomics have been performed. North *et al*. reported the *N*-glycomic profiles of parental Chinese Hamster Ovary (CHO Pro-5) and glycosylation mutant (CHO Lec1, CHO Lec2, *etc*.) cells [29]. In this study, cell pellets were sonicated, reduced and carboxymethylated and then digested with trypsin. Permethylated purified glycans were subjected to MALDI-TOF MS. This analysis revealed an unexpectedly large complexity of *N*-glycans in CHO cell mutants. The *N*-glycosylation profile of mouse and human immune cells, assessed by the same approach, has also been reviewed recently [30]. Satomaa *et al*. were the first to report the *N*-glycomic profile of human ES cells and their differentiated progeny [31]. In this study, cell pellets were subjected to PNGase F digestion and then purified by sequential precipitation/extraction using C18 silica chromatography columns with strong cation-exchange resin, porous graphitized carbon and microcrystalline cellulose (for the sialylated glycans). The purified *N*-glycans were directly subjected to MALDI-TOF MS analysis without any derivatization. This analysis revealed statistical significance for the human ES cells-associated *N*-glycan signals, e.g., large high-mannose type *N*-glycan signals, Man7, Man8 and Man9, as well as complex fucosylated *N*-glycan signals, (Hex)_5_(HexNAc)_4_(Fuc)_2_, (NeuAc)_1_(Hex)_5_(HexNAc)_4_(Fuc)_2_ and (NeuAc)_1_(Hex)_5_(HexNAc)_4_(Fuc)_3_. This study also demonstrated that the *N*-glycan phenotype of human ESs reflected their differentiation stage. Notably, the authors of the study analyzed the glycans by 800 MHz ^1^H nuclear magnetic resonance (NMR) using cryo-probes.

Furukawa *et al*. captured glycans with a solid-phase hydrazide-functionalized glycoblotting polymer (BlotGlyco H), followed by washing to remove the non-bound material [32]. Unreacted hydrazide groups were blocked with acetic anhydride, and sialic acids were stabilized by methyl ester formation using 3-methyl-p-tolyltriazine [21]. The blotted glycans were recovered in their reducing terminal-derivative form by adding the aminooxy-containing compound aoWR, which substantially improves detection sensitivity with MALDI-TOF MS analysis [33]. This procedure can be performed in a 96-well plate format and can be used with MALDI-TOF MS for *N*-glycomic analysis of murine dermis and epidermis [34], as well as murine ES cells and their differentiated progenies (*i.e.*, cardiomyocytes or neural cells) [35].

**Scheme 1:**
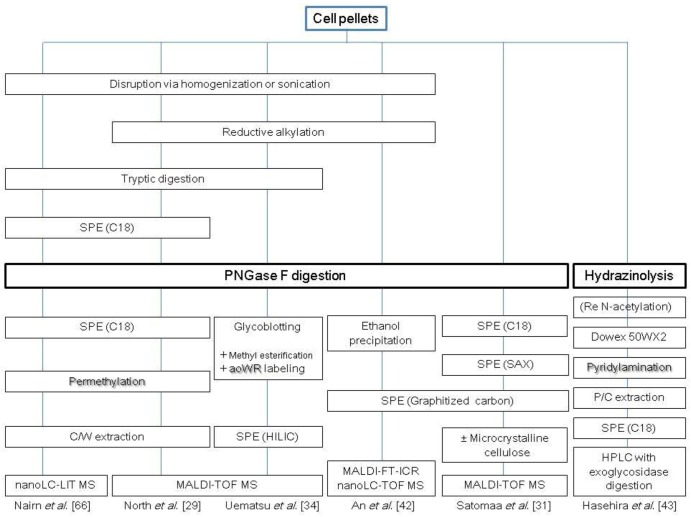
Representative protocols for cellular *N*-glycomic analysis. C, chloroform; M, methanol; W, water; P, phenol; SPE, solid-phase extraction.

Chromatographic profiling of glycans enables not only the discovery of structure-specific markers, but also unprecedented structural analysis of the glycome. Coupling of chromatographic profiling with MS complements the strategy of glycan mass profiling by MALDI-TOF MS, since MALDI-TOF MS profiling alone is often unable to discriminate isomers and detect low abundant glycan species due to its limited dynamic range (typically in the order of 10^2^ to 10^3^). Separation modes for *N*-glycan analysis include reversed-phase (RP) chromatography [36,37], normal-phase chromatography/hydrophilic interaction chromatography (HILIC)[38,39] and graphitized carbon chromatography [40], all of which display good compatibility with MS and high resolution power. MS analyzers, such as ion trap, quadrupole, FTICR orbitrap and ion mobility with electrospray ionization have been used for glycomic and glycoproteomic analysis [41]. An *et al*. reported the *N*-glycomic profile of enriched plasma membranes from human ES cells [42]. In combination with MALDI-TOF MS analysis, this study analyzed *N*-glycans using a microfluidic HPLC-ChiP-TOF MS system to discriminate isomers. The microfluidic HPLC-Ch consisted of an enrichment column, an LC separation column packed with porous graphitized carbon and a nanoelectrospray tip. The microchip was interfaced with a TOF mass analyzer that routinely provides a mass measurement accuracy of less than 5 ppm. The isolated membrane fraction was reduced with dithiothreitol (DTT) and then subjected to PNGase F digestion. Following ethanol precipitation, *N*-glycans were purified by solid-phase extraction (SPE) using a graphitized carbon cartridge. This study identified an average of 170 distinct features, which included anomers and other isomers, arising from an average of 69 glycan compositions. Hasehira *et al*. reported the *N*-glycan profiles of a human iPS cell line and human dermal fibroblasts [43]. In this study, the glycans were liberated by gas-phase hydrazinolysis, fluorescently tagged with 2-aminopyridine (PA) at their reducing terminus, and then, the derived PA-glycans were purified by multiple-mode (anion-exchange, size-fractionation and RP) high-performance liquid chromatography (HPLC). The glycan structures were determined and quantified by HPLC mapping with MALDI-TOF-MS and exoglycosidase digestion analyses. It was found that the type of linkage of sialic acid (Sia) on *N*-linked glycans was dramatically changed from α-2-3 to α-2-6, and the expression of α-1-2 fucose and type 1 LacNAc structures became clearly and apparently increased upon induction of pluripotency. Several representative strategies for the analysis of cellular *N*-glycans are summarized in Scheme 1. 

Analysis of extensive *N*-glycosylation sites can be performed using hydrazide-capture methods, as described previously [44]. Briefly, glycoproteins are chemically oxidized with sodium peroxidate and captured on hydrazide beads. The unbound non-glycosylated proteins are removed by rinsing, and the captured glycoproteins are subjected to on-bead reduction, alkylation, denaturation and tryptic digestion. The bound peptides are then labeled with stable isotope reagents for quantification, and the *N*-glycosylated peptides are released with PNGase F, purified by RP-SPE and analyzed by LC-MS/MS. This method has been utilized to elucidate the *N*-glycosylation site of multiple cell types, including CHO cells [45], human bronchial epithelial cells [46] and a human pancreatic carcinoma cell line [47]. Kurogochi *et al*. described a strategy for analysis of sialic acid-containing glycopeptides that involves selective oxidation of sialic acid residues to elaborate terminal aldehyde groups and subsequent enrichment by chemical ligation with hydrazide beads [48].

Recent progress in MS techniques, including electron capture dissociation (ECD) and electron transfer dissociation (ETD) fragmentation, have enabled analysis of both glycan and peptide moieties, thus providing a powerful measurement tool for *N*-glycoproteomics [49]. However, extensive direct analysis of the cellular *N*-glycoproteome that unveils both glycan and peptide moieties is still a challenging task, due to the tremendous heterogeneity generated by both glycan and peptide moieties. Despite this difficulty, once target glycopeptides are identified, multiple reaction monitoring allows quantification of glycopeptides from complex biological samples [48,50].

### 2.2. *O*-Glycans

*O*-glycosylation represents a very diverse group of modifications, including the *O*-GalNAc type typical of mucin and the *O*-GlcNAc, *O*-Fuc, *O*-Man and *O*-Xyl types [51]. These modifications not only activate proteins, but also serve as markers indicating binding to other proteins. *O*-GlcNAcylation involves a single *O*-GlcNAc residue, while other modifications subsequently form more complicated oligosaccharide structures through the actions of various glycosyltransferases. Site-specific *O*-glycosylation is an important regulator of protein function; the most recently discovered function of *O*-glycosylation is co-regulation of protein processing by proprotein convertase [52,53]. The dynamic cell- and protein-specific regulation of site-directed glycosylation can be essential for development and health [52,54,55]. The initiating event of *O*-glycan biosynthesis that is classified into the mucin type is transfer of monosaccharidic GalNAc (from UDP-GalNAc) to serine and threonine residues via catalysis by a polypeptide GalNAc transferase. The resultant protein-linked GalNAc can be extended into different structural core classes (core 1 to core 8) by various glycosyl transferases. Mucins are high molecular weight glycoproteins characterized by a high density of glycans that form complex macromolecular structures [56,57]. Changes in mucin *O*-glycosylation appear to be a general phenomenon of malignancies [58,59]. The *O*-glycopeptide of MUC1 has been targeted in cancer and may be of diagnostic value [60].

Although the *N*-glycans in glycoproteins can be released by specific enzymes, such as PNGase F and PNGase A [14,61], *O*-glycan release is limited because an analogous endoglycosidase is currently unavailable. The commercially available endo-α-*N*-acetylgalactosaminidase (*O*-glycanase) has a high substrate specificity and is used only for the removal of core 1 disaccharides (Galβ1-3GalNAc) from mucin glycoproteins [62]. Therefore, most *O*-glycans are released from the core proteins by chemical procedures. Various chemical digestion approaches are often accompanied by significant loss of the intact *O*-glycans, due to serious glycan degradation (referred to as peeling reactions). Over the last two decades, several chemical release procedures have been studied. In the Human Proteome Organization’s human disease glycomics/proteomics initiative, *O*-glycans in a number of glycoprotein samples were analyzed in 15 laboratories worldwide [63]. Unlike the same analysis of *N*-glycans, where fairly consistent results were obtained among different laboratories and techniques, the results obtained for *O*-glycans differed substantially among the laboratories and techniques, suggesting that there continues to be a strong and growing need for sensitive, rapid and highly quantitative analysis of *O*-glycans. However, recent rapid progress in *O*-glycan analysis has begun to allow clarification of cellular *O*-glycomics.

Reductive β-elimination, reported in 1968 by Carlson [64], is still the most reliable technique for liberation of *O*-glycans and has proven to be a feasible method for cellular *O*-glycomic analyses. Reductive β-elimination releases *O*-glycans in their reduced form (alditol form) as a result of immediate *in situ* reduction by sodium borohydride. Reduction of the innermost sugar to alditol minimizes the side peeling reaction. One limitation of this method is that reduction causes a loss of the reducing end of the carbohydrate, which precludes additional downstream glycomic analysis, such as derivatization suitable for enrichment, chromatographic and mass spectrometric analyses. Using the reductive β-elimination technique combined with MALDI-TOF analysis, Babu *et al*. reported the structural characterization of neutrophil *O*-glycomics [65]. Glycoproteins from human neutrophil cells were subjected to reductive carboxymethylation followed by digestion with trypsin. *O*-glycans were then released from tryptic glycopeptides by reductive β-elimination, and purified glycans by Dowex 50W-X8 were permethylated to enhance the sensitivity of MS detection. The *O*-glycomic profile consisted of core 1 and core 2 *O*-glycans with sialyl Le^x^ and Le^x^ as non-reducing terminal epitopes. The same technique was also used to determine the *O*-glycomic profile of parent and glycosylation mutant CHO cell lines [29]. The *O*-glycans from both wild-type (parent) and mutant CHO cell lines showed a simple set of core 1 structures comprising T-antigen, sialylated T and di-sialylated T. Nairn *et al*. reported the *O*-glycomic alteration of mouse ES cells upon differentiation into embryoid bodies or extraembryonic endodermal cells [66]. In this study, 28 types of *O*-glycans were identified, including mannosyl-, fucosyl- and glucosyl-type *O*-glycans. The authors of this study concluded that changes in glycan structures correlated with alterations in transcript abundance of the corresponding biosynthetic enzymes, suggesting that transcriptional regulation contributes significantly to the regulation of glycan expression. Maniatis *et al*. reported microwave-assisted reductive β-elimination using dimethylamine, which enabled short-time quantitative release of *O*-glycans from model glycopeptides and glycoproteins [67].

Extensive attempts to establish non-reductive β-elimination methods have been made, with the aim of enabling subsequent derivatization of reducing terminal for high sensitive detection. Huang *et al*. reported ammonium-based alkali-catalyzed non-reductive β-elimination [68], though it was reported that peeling cannot be completely prevented, and the hydrolysis yield is low compared to the reductive β-elimination method [69]. Natunen *et al*. used the technique to identify the *O*-glycomic profile of human ES cells [7]. This study identified (Hex)3(HexNAc)3, which has a Galβ1-3GlcNAc epitope at the non-reducing end and is tentatively assigned to be Galβ1-3GlcNAcβ1-3Galβ1-4GlcNAcβ1-6 (Galβ1-3GlcNAcβ1-3)Galβ1-4Glc, They reported that this structure is the epitope for Tra-1-60 and Tra-1-81 antibodies. 

**Scheme 2:**
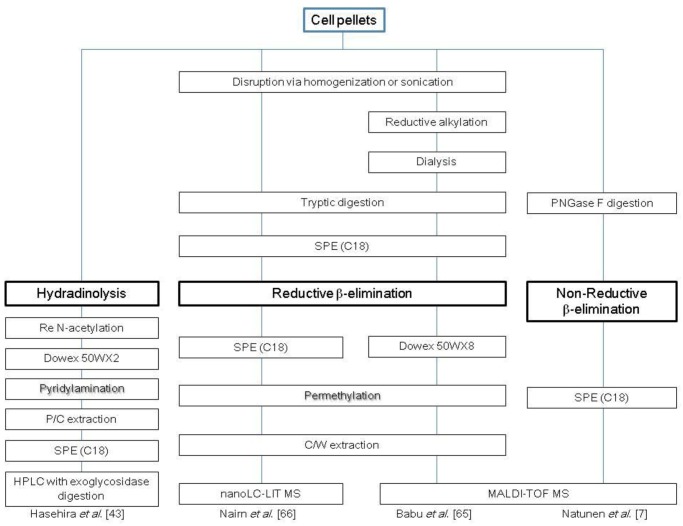
Representative protocols for cellular *O*-glycomic analysis. C, chloroform; M, methanol; W, water; P, phenol; SPE, solid-phase extraction.

Hydrazinolysis is another chemical de-*O*-glycosylation method that produces *O*-glycans in a non-reduced form [70,71,72,73,74]. Hasehira *et al*. used this method to report the *O*-glycomic profile of human iPS cells and human dermal fibroblasts [43]. A cell pellet generated from approximately 1 × 10^7^ cells was treated with anhydrous hydrazine at 100 °C for 4 h, and the released glycans were re-*N*-acetylated. The resulting non-reductive *O*-glycans were fluorescently tagged with 2-aminopyrydine and then analyzed using HPLC mapping assisted by MALDI-TOF-MS and exoglycosidase digestion. This study identified a total of ten different types of *O*-glycans and concluded that *α*-1-2 linked fucosylation and type 1 LAcNAc structures are critical epitopes that are characteristic to iPS cells. Several representative strategies for the analysis of cellular *O*-glycans are summarized in Scheme 2.

Zauner *et al*. [75], Wang *et al*. [76] and Furukawa *et al*. [77] have introduced a novel one-pot *O*-glycome analytical method combined with release from glycoproteins and labeling with pyrazolone analogs. This method, namely β-elimination in the presence of pyrazolone analogs (BEP), allows simultaneous labeling of released *O*-glycans with pyrazolone analogs and, thus, minimizes the undesirable peeling reaction. 

Identification of *O*-glycosylation sites can be performed by β-elimination and subsequent Michael addition (BEMA). Hedou *et al*. identified the *O*-GlcNAc modification sites in purified contractile protein homogenates using an MS-based method that relied on BEMA of DTT, following dephosphorylation to discriminate between phosphorylation and *O*-glycosylation sites [78]. BEP allows analysis of released *O*-glycans and formerly *O*-glycosylated glycopeptides, because both species can be labeled by pyrazolone analogs and are thus amenable to further analysis [77]. It was recently reported that *O*-GlcNAc regulates pluripotency and reprogramming by acting directly on core components of the pluripotency network [79]. Oct4 was purified by immunoprecipitation of ZHBTc4 F-Oct4 cell lysates with a Flag antibody. *O*-GlcNAcylated Oct4 was then enriched by WGA pull down. After SDS-PAGE and Colloidal Blue Staining, the Oct4 band was analyzed using nano-LC-ESI-MS/MS. Trinidad *et al*. identified greater than 1,750 and 16,500 sites of *O*-GlcNAcylation and phosphorylation, respectively, by using a combination of fragmentation techniques, including ECD, collision-induced dissociation (CID) and higher-energy collisional dissociation (HCD) for GlcNAcylated, phosphor and non-modified peptides, respectively [80]. A technique for extensive *O*-glycosylation that uses an enzymatically glyco-engineered human cell line (SimpleCell line) expressing homogenous truncated *O*-glycosylation with GalNAcα (Tn) or NeuAcα2-6GalNAcα (STn) *O*-glycans was recently reported by Steentoft *et al*. This technique allows straightforward isolation and sequencing of GalNAc *O*-glycopeptides from total cell lysates using lectin chromatography and nanoflow liquid chromatography-MS [81]. In total, greater than 100 *O*-glycoproteins with greater than 350 *O*-glycan sites were identified. 

Recent progress in MS techniques, including ECD and electron-transfer dissociation (ETD) fragmentation, have enabled analysis of both glycan and peptide moieties; these methods have thus provided very powerful measurement tools for *O*-glycoproteomics [82]. However, extensive direct analysis of cellular *O*-glycoproteomics, unveiling both glycan and peptide moieties, is still a difficult task, due to the tremendous heterogeneity generated by these moieties.

### 2.3. Glycosphingolipids

Glycolipids are lipid molecules linked to one or more carbohydrate units. Based on their lipid moiety, glycolipids are classified as either glycosphingolipids (GSLs), containing ceramide, or glycoglycerolipids, containing glycerol. In mammals, glycolipids are predominantly present as GSLs [83]. GSLs are one of the major components of plasma membrane lipid rafts and play important roles in various biological events, such as cell-cell interactions, signal transduction and cellular differentiation [84,85,86,87,88,89,90,91]. The structural classification of GSLs is defined by their glycan structure rather than by their ceramide moieties and includes ganglio-, globo-, isoglobo-, lacto- and neolacto-series. Among these classes, globo-series GSLs contain a treasure trove of undifferentiated cell markers, such as SSEA-3 (Gb5) and SSEA-4 (sialyl Gb5), which were first isolated from teratocarcinoma [5] and form Galβ1-3GalNAcβ1-3Galα1-4Galβ1-4Glcβ1-Cer and NeuAcα2-3Galβ1-3GalNAcβ1-3Galα1-4Galβ1-4Glcβ1-Cer, respectively. Moreover, globo H GSL (fucosyl Gb5), which contains a type IV H-antigen with a Fucα1-2Galβ1-3GalNAcβ1-3Galα1-4Galβ1-4Glcβ1-Cer structure, was also identified as a promising undifferentiated cell marker in studies using breast cancer stem cells [92] and human ES cells [93]. Other cell-specific GSLs, such as cancer-associated gangliosides (e.g., GD2, GD3, GM2, fucosyl GM1 and Neu5GcGM3) have also been reported and are expected to be promising antigens for the development of cancer vaccines [94]. Furthermore, several human cluster of differentiation (CD) markers, such as CD17 (LacCer), CD60a (GD3), CD60b (9-O-acetyl GD3), CD60c (7-O-acetyl GD3) and CD77 (Gb3), are comprised of GSLs [95]. 

Among various glycoconjugates, glycomic analyses of GSLs have been performed extensively using thin layer chromatography (TLC) since the mid-1960s. Recently, MS analysis has played a principal role in the structural and quantitative analysis of GSLs. Of the two major approaches, which include analysis of the intact form of GSLs and analysis of the glycan moiety upon deglycosylation, the former approach provides valuable information regarding both glycomics and lipidomics simultaneously. 

GSLs are generally extracted from cells by homogenization in a solvent consisting of chloroform, methanol and water. Soluble GSLs are recovered from the supernatant by centrifugation. The GSL fraction may be further fractionated into polar and nonpolar GSLs by phase partitioning. Because glycerolipids sometimes interfere with the analysis of GSLs, these compounds are occasionally saponificated under mild alkaline conditions [96]. The extracted and purified GSLs are usually permethylated prior to MALDI-TOF MS analysis [97]. Combination of MS with TLC or LC circumvents the inadequacies of MS analysis alone, such as separation of structural isomers, and increases the quantitative capability of the glycosphingolipidomic technique [98,99,100]. Liang *et al*. used MALDI-MS(/MS) and gene expression profiling of glycosyltransferases involved in GSL synthesis to identify a drastic structural alteration of cellular GSLs from globo- and lacto-series to ganglio-series during the differentiation of human ES cells to embryoid bodies [93]. MS analyses of GSL-associated glycans that are targeting cancer diagnosis and therapy are also currently in progress. Gupta *et al*. performed a comparison of GSL-derived glycan structures in cancer stem and non-stem cells and showed that enhanced ceramide glycosylation and the expression level of Gb3 correlate well with the numbers of cancer stem cells in breast cancer cell lines [101]. This result indicates that GSL-glycan profiling has a high potential for determination of the malignancy grading of cancer and for cancer therapy. 

Liquid chromatography is useful to discriminate isomers (e.g., GlcCer and GalCer) and to detect low abundant glycan species to reduce ionization suppression. RP chromatography is used for separations based on the length and saturation of acyl chains and normal phase chromatography to separate compounds primarily by their headgroup constituents (for example, distinguish Cer, GlcCer, LacCer, globotriaosylceramide, globotetraosylceramide, sphingomyelin, as well as cholesterol, *etc*.) [102]. Microfluidic or nanofluidic LC paired with ESI-MS analysis with multiple reaction monitoring (MRM) can be used to quantify GSLs. Ikeda *et al*. reported a highly effective method using LC/ESI-MS/MS with MRM for quantitative structural analysis of ganglio-series GSLs and sulfatides extracted from mouse brain [103] and mouse cerebellum sections [104].

Another cellular GSL glycomics approach is the analysis of the glycan moiety following release of the head-group glycans from ceramide. By focusing on the glycan moiety alone, it is possible to reduce the complexity of GSLs that arises from the heterogeneity generated by both glycan and ceramide moieties. The oligosaccharides can be detached from GSLs by either enzymatic or chemical digestion. The enzymatic treatment involves the use of endo-type glycosylceramidases (EC 3.2.1.123) that specifically cleave the linkage between glycan and ceramide. These enzymes, called ceramide glycanases (CGases) or endoglycoceramidases (EGCases), have been isolated from some invertebrate species and bacteria [105,106,107,108,109,110,111]. Enzymatic digestion allows analysis of both glycan and ceramides and, therefore, enables simultaneous glycomic and lipidomic analyses. However, one limitation of this approach is the relatively high substrate specificities of endo-type glycosylceramidases. Fujitani *et al*. reported maximal deglycosylation efficiency by using a mixture of Rhodococcal EGCase I and II [108,109]. In this study, the GSL fraction, which was recovered by traditional homogenization of cells in a solvent consisting of chloroform and methanol, was subjected to Rhodococcal EGCase I and II digestion. The liberated GSL glycans were directly purified by glycoblotting, and their reducing termini were simultaneously labeled with aoWR. The labeled glycans were then subjected to MALDI-TOF MS [112]. The cellular GSL glycomic profiles of 11 different types of cells, including embryonic carcinoma cells, were studied. This study demonstrated that both the relative proportion of GSL glycans and the total amount of expressed GSL glycans are highly cell-specific and, therefore, provide a novel measure for the characterization of cells. Mesenchymal stem cells (MSCs) are an invaluable tool for disease therapy and regenerative medicine studies. Glycomic characterization, including identification of GSL-associated oligosaccharides from bone marrow-derived MSCs, has been performed using a combination of MALDI-MS(/MS), NMR and glycosidase digestions [113]. Glycosphingolipid glycans were detached by *Macrobdella decora* endoglycoceramidase digestion. The glycome profiles of MSCs and osteoblast cells that differentiate from them displayed distinct differences, indicating that glycosylation analyses can be used to evaluate the differentiation state of MSCs.

**Scheme 3:**
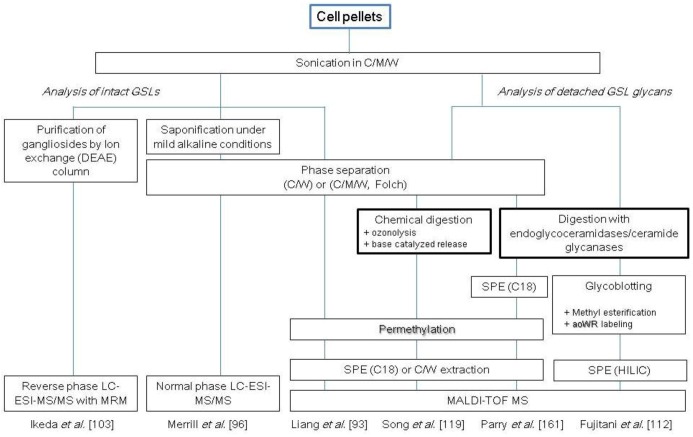
Representative protocols for the analysis of cellular glycosphingolipids (GSLs) and GSL-linked glycans. C, chloroform; M, methanol; W, water; SPE, solid-phase extraction.

Another deglycosylation method is based on chemical digestion, which is traditionally performed by ozonolysis or osmium-catalyzed periodate oxidation to cleave the olefinic double bond of sphingosine, followed by alkaline treatment to release glycans by a β-elimination-like reaction [114,115,116,117,118]. The major advantage of chemical digestion is the non-specific cleavage of glycan from GSLs, which allows recovery of multiple types of glycan head-groups. To date, few studies have employed chemical digestion for glycomic analyses of cellular GSLs. Song *et al*. recently reported a novel method to release glycans from GSLs under near neutral pH conditions; this method drastically suppresses the peeling reaction, as demonstrated by analysis of the GSL-derived glycans from human erythrocytes [119]. Several representative strategies for the analysis of cellular GSLs and GSL-linked glycans are summarized in Scheme 3.

### 2.4. Glycosaminoglycans

Proteoglycans (PGs) are a diverse group of glycoconjugates consisting of various core proteins that are post-translationally modified with linear, anionic polysaccharide glycosaminoglycans (GAGs) consisting of repeating disaccharides. PGs are distributed ubiquitously throughout the extracellular matrix and are found on virtually all cell surfaces and basement membranes of different tissues. Through their interaction with proteins, PGs mediate numerous biological processes, including cell-cell and cell-matrix interactions, growth factor sequestration, chemokine and cytokine activation, regulation of stem cell differentiation, tissue morphogenesis during embryonic development, cell migration and cell proliferation [120,121].

Characterization of PGs is challenging due to the tremendous structural diversity of the bioconjugates that arise from multiple isoforms of PG core proteins and variations of their GAG components, although it is noteworthy that analysis of the GAGs is much more difficult than analysis of the core proteins. All GAGs consist of repeating disaccharide structures. The simplest GAG is hyaluronic acid (HA); this non-sulfated free polysaccharide, which is synthesized at and extruded from the plasma membrane, consists of [Galβ1-3GlcNAcβ1-4]_n_ with n > 2,000 in extracellular matrices. Heparin and heparan sulfate (HS) chains are synthesized as [GlcAβ1-4GlcNα1-4]. The GlcN moiety may be 6-*O*-sulfated, *N*-sulfated, *N*-acetylated or sporadically 3-*O*-sulfated; the GlcA moiety may be epimerized to iduronic acid (IdoA) ; the IdoA may be sulfated at the 2-position. Chondroitin sulfate (CS) consists of repeating units of [GlcAβ1-3GalNAcβ1-4] and may be sulfated at the 4- and/or 6-position of GalNAc. Dermatan sulfate (DS) is a CS variant in which a substantial fraction of GlcA residues are epimerized to IdoA; the IdoA may be sulfated at the 2-position. HS, CS and DS are bound to serine (Ser) residues of proteoglycan core proteins via a [GlcAβ1-3Galβ1,3Galβ1,4Xylβ1-O-Ser] tetrasaccharide linker. Keratan sulfate (KS) is a sulfated polylactosamine chain consisting of repeating units of [Galβ1,4GlcNAcβ1,3]. KS consists of approximately 50 disaccharide residues and is linked to the protein either as an extension to an *N*-linked glycan (KS I) or through a Ser/threonine (Thr) bound linker structure (KS II) [122].

Ly *et al*. reported the first direct analysis of purified peptidoglycan (pG) prepared from bikunin, the simplest PG having one CS chain, by MS and CID-MS/MS experiments with both Fourier transform ion cyclotron resonance (FT-ICR) and Fourier transform mass spectrometry (FTMS) instruments [123]. However, structurally intensive analysis of intact PGs has been a virtually impossible task to date, because of the tremendous structural diversity of these molecules. The fine structures of GAGs are assessed by analyzing the disaccharide composition, which typically involves isolation of PGs, recovery of the GAG fraction, enzymatic depolymerization, labeling of disaccharides and then determination of the disaccharide composition.

Due to the large hydrodynamic radius and high net negative charge of linear GAG chains, isolation of PGs is a fairly straightforward task. A protocol for the recovery and purification of GAGs established by Linhardt and coworkers consists of homogenization of cells, delipidation, proteolysis of PGs by nonspecific protease (e.g., actinase E) to obtain pGs, extraction of pGs into CHAPS/urea and then recovery of pGs by spin column-based ion chromatography and subsequent membrane-based desalting [124]. Guimond *et al*. developed a rapid and efficient method for PG extraction in which exclusive partition of PGs into the aqueous phase of TRIzol-chloroform can be achieved in less than one hour [125]. The resulting extract can be applied directly to the anion-exchange medium, which eliminates the requirement for a buffer exchange step to urea. Anion-exchange chromatography is effectively utilized for the purification of GAGs in both cases. Non-sulfated GAGs in biological samples, such as HA and *N*-acetyl heparosan, are often ignored or underestimated. Zhao *et al*. employed mini strong anion-exchange spin columns with an optimized concentration of sodium chloride solution to enable high recovery of non-sulfated GAGs [126]. A protocol recently reported by Takegawa *et al*. consists of homogenization of cells, delipidation, proteolysis by Pronase and then ethanol precipitation. The relatively low purification efficiency, which is possibly caused by eliminating the anion-exchange process, is compensated by the employment of chemoselective glycoblotting for purification of glycans. Since this protocol does not rely on ion exchange chromatography, the loss of non-sulfated GAGs is reduced [127].

Purified pGs can be subjected to PAGE or gel filtration chromatography for fractionation and molecular weight determination of GAGs [124,128]. For disaccharide analysis, GAGs are usually depolymerized by commercially available GAG lyases, which cleave a HexNAc-HexA bond and leave a C4-C5 unsaturated HexA at the new non-reducing terminus. Chondroitinase ABC from *Proteus vulgaris* is a nonspecific lyase that cleaves all forms of CS. A combination of three heparin lyases (Heparin lyase-I, II and III) from *Flavobacterium heparinum* is often used for HS-PG glycomic studies [129]. In the case of KS, keratanase (from *Pseudomonas*) and keratanase II (from *Bacillus* sp. Ks36) are used; these enzymes act via a hydrolytic mechanism to produce saturated residues. Keratanase is an endo-β-1,4-galactosidase that requires an unsulfated galactosamine (Gal) residue flanked by a 6-*O*-sulfated GlcNAc residue; keratanase II is an endo-β-1,3-glucosaminidase that requires sulfation at the 6-*O* position of GlcN and thus cleaves at a majority of 6-*O*-sulfated GlcNAc residues. HA is typically converted with hyaluronidase SD (from *Streptococcus dysgalactiae*) to an unsaturated disaccharide. The depolymerized products are usually purified by ultrafiltration [124] or diethylaminoethyl (DEAE) chromatography followed by ultrafiltration [125].

Since a C4-C5 unsaturated double bond is introduced at the non-reducing end of the uronic acid residue upon lyase treatment, direct measurement of absorbance at 232 nm is possible [123,130]. To enable more sensitive detection, the products of GAG depolymerizing enzymes are often labeled with fluorescent tags, such as 2-aminoacridone (AMAC) [131,132], 2-aminobenzamide (2-AB) [133], 2-aminobenzoic acid [134] or boron-dipyrromethene (BODIPY) hydrazide [135], which target newly created reducing ends. The use of isotope-coded tags for MS detection, including [^12^C_6_]/[^13^C_6_] anilines [136], 2-anthranilic acid-d_0_ or -d_4_ [137] and tetraplex stable isotope-coded tags [138], has also been reported. Unlike the analysis of *N*-, *O*- and GSL-glycans, where MALDI-TOF MS is often the analytical technique chosen, the separation process is crucial for GAG disaccharide analysis, since many of constituent disaccharides share identical charge and mass. Though several separation techniques, including capillary electrophoresis (CE) [139,140], fluorophore-assisted gel electrophoresis (FACE) [141] and HPLC, have been used for disaccharide compositional analysis, HPLC is the most popular method for cellular GAG disaccharide analysis, due to its general versatility and variety of useful separation modes. The application of ultra-performance liquid chromatography (UPLC) performed at high pressure (up to 10^8^ Pa) with 1.7 µm particles and a proprietary mobile phase pump provides shorter separation times with improved separation [142].

Strong anion-exchange HPLC has conventionally been used to separate disaccharides; however, interfacing this technique with MS is difficult [143]. RP ion-pairing (IP) HPLC, which employs volatile primary, secondary and tertiary amines as post-column additives, provides excellent chromatographic resolution and is compatible with MS detection [144], although the IP reagents seriously contaminate the MS ion source. HILIC separates glycans based on their size and number of acidic groups under solvent conditions appropriate for LC/MS [145]. Takegawa *et al*. recently reported simultaneous analysis of 2-AB labeled disaccharides from HS/HP, CS/DS and HA by single-step zwitterionic-hydrophilic interaction chromatography (ZIC-HILIC-HPLC) [127]. Volpi reported the analysis of CS/HA disaccharides by employing labeling with 2-aminoacridone (AMAC) and RP chromatography [146]. Yang *et al*. amended this analytical protocol to enable analysis of 17 AMAC-tagged disaccharides from HS/HP, CS/DS and HA by a single RP-UPLC-MS experiment [126,147]. These studies are the first successful demonstrations of simultaneous analysis of all three families of uronic acid-containing GAGs by a single chromatographic method. Due to their acidity, GAG oligosaccharides are usually analyzed by ESI-MS in the negative ionization mode. The topic of MS analyses of GAGs has been extensively reviewed elsewhere [41,148].

By utilizing the methods described above, GAG disaccharide profiles have been unveiled for many cells, including embryonic-like tetracarcinoma cells [124], mesenchymal cells [149,150,151], murine ESs [152], as well as various other human cells and cell lines from other animal species [127,128,136]. Lawrence *et al*. reported the HS and CS disaccharides and KS digestion products of various cells, including mutant and wild-type CHO cells and other mammalian cells [136]. GAG chains were extracted from cell pellets by exhaustive digestion with Pronase followed by AEC (DEAE-Sepharose). pGs were digested with GAG degradation enzymes, and the resultant HS disaccharides, CS disaccharides and KS digestion products were labeled with [^12^C_6_]/[^13^C_6_] anilines, which were then analyzed using RP-IP-ESI-MS. This study demonstrated significant qualitative and quantitative differences in the structures of GAGs among different organisms. Nairn *et al*. reported the first HS, CS/DS and HA-GAG disaccharide profiles of murine ESs and their differentiated cells by RP-IP-HPLC with post-column fluorescence detection [152]. This group found that the overall GAG content increased and the sulfation patterns changed as ESs differentiated into embryoid bodies and extraembryonic endodermal cells. The same approach was also used to profile human embryonic-like tetracarcinoma cells [124]. Kawabe *et al*. characterized the epitope of a novel monoclonal antibody (R-10G) specific to human iPS/ES cells by KS glycomic analysis of human iPS cells, which led to the conclusion that the R-10G epitope is a unique KS that lacks oversulfated structures [153]. Several representative strategies for the analysis of cellular GAG disaccharides are summarized in Scheme 3.

Further structural elucidation of PGs/GAGs can be performed by partial enzymatic or chemical depolymerization under controlled conditions [154]. In studies involving PG core protein sequencing or identification, GAGs can be released from their Ser attachment sites using BEMA, as described earlier in this review (refer to “*O*-glycome” section). The GAG-protein linkage region tetrasaccharide (GlcAβ1-3Galβ1-3Galβ1-4Xylβ1-O-Ser) is the subject of many studies, owing to its proposed role in the initiation of GAG chain biosynthesis. Modifications of the linkage region tetrasaccharide, such as 2-*O*-phosphorylation of Xyl or 4-*O*-sulfation and 6-*O*-sulfonation of Gal residues, have been correlated with changes in the number and type of GAG chains present in PGs [155]. Lawrence *et al*. reported a novel approach based on the detection of nonreducing ends of the glycosaminoglycans that accumulate in cells, blood and urine of individuals with mucopolysaccharidoses (MPS). After GAG depolymerization, the specific nonreducing ends fragments of the GAGs that are unique in each MPS are quantified [156].

**Scheme 4:**
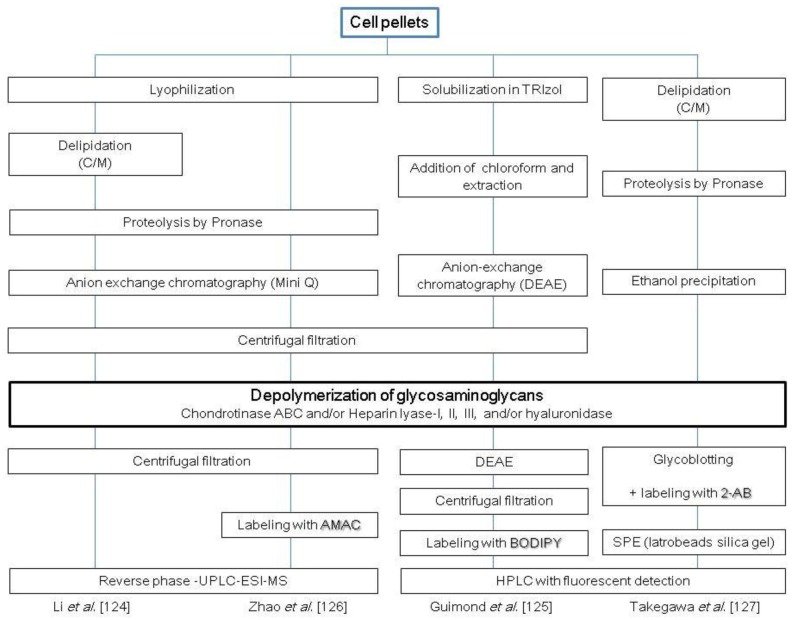
Representative protocols for cellular glycosaminoglycans (GAG) disaccharides analysis. C, chloroform; M, methanol; W, water; SPE, solid-phase extraction.

## 3. Perspective

This review has described the recent advances in elementary glycomic analysis of *N*- and *O*-linked glycans derived from glycoproteins, proteoglycans and glycosphingolipids, with a special focus on their application to cellular glycomics. An understanding of the elementary glycome is an important prerequisite for extensive cellular glycomics and precise cellular characterization. To date, reports of extensive cellular glycosylation analyses have been scarce. Wearne *et al*. employed fluorescently-labeled lectins to classify structural glycosylation motifs on the stem cell surface [157], and the utility of lectin arrays for cell characterization has recently been demonstrated [72,158,159]. Lectin arrays are shown to be capable of profiling unique bacterial cell surface glycomes in part [160]. Glycosylation profiling based on carbohydrate-recognizing molecules, such as lectins and antibodies, provides valuable information about sugar epitopes. However, only a limited number of lectins and glycan-specific antibodies are currently available, and it is often difficult to discriminate between different glycoconjugate species by these methods. In this regard, glycomic analyses based on molecular recognition and structurally intensive studies using sophisticated analytical chemistry (e.g., separation techniques, MS, *etc*.) are complementary. This review focused on the structurally intensive studies. Analysis of the comprehensive cellular glycome has been addressed in pioneering MS work. Parry *et al*. reported a mass spectrometric strategy to characterize both glycans from GSLs and glycoproteins using mouse brain as a model case [161]. Heiskanen *et al*. unveiled *N*- and *O*-linked glycans derived from glycoproteins and glycans of GSLs of bone marrow-derived MSCs by MS [113]. 

No attempts have yet been made to determine all major classes of glycans or the relative concentration of each type of glycan present within a cell. Integration of elementary glycomic analyses to obtain a comprehensive understanding of the glycome requires streamlining the sample preparation protocol as effectively as possible. Development of techniques that enable multiplexing is also required to improve the throughput of glycomic studies, as has been achieved for proteomic and transcriptomic analyses. To compare the relative concentration of each type of glycan derived from various sources of glycoconjugates, absolute quantification, rather than relative quantification, is most important. The number of cells required for the glycomic analyses reviewed in this article is typically in the range of 10^4^ and 10^8^ cells, depending on the specific technique employed and the type of glycoconjugate. Since detection sensitivity is defined by signal-to-noise ratio, it is important to reduce the noise as much as possible, which may be achieved through an optimized sample preparation technique. Increasing the signal may be achieved by employing an optimized labeling strategy and/or highly sensitive analytical technique. Current glycomic analytical protocols do not allow analysis of species with low abundance, which is another issue that needs to be addressed. Another issue to be addressed is the necessity of informatics to link and interpret the data obtained by these different methods.

A panoramic view of the extensive cellular glycome has multiple potential applications. For example, comprehensive identification of pluripotency biomarkers and disease-related biomarkers will be accelerated. In addition, elucidation of the overall picture of cellular glycosylation and its alteration upon differentiation or acquisition of disease would provide a useful foundation for analysis of iPS and differentiated cells during drug discovery studies. An understanding of the entire cellular glycome is also expected to expedite the development of more specific biomarkers that can discriminate between somatic stem cells and cancerous cells, as well as cancer vaccines for which identification of a specific antigenic epitope is most important. Analysis of all major glycans, including those described in this review, as well as other glycans/glycoconjugates, such as GPI anchors, intracellular lipid-linked glycans, free cellular oligosaccharides and sugar nucleotides, will allow deciphering of intra- and inter-glycomic correlations. Overall, the ability to analyze the cellular glycome extensively and completely will open new avenues in systems biology glycomics.
